# Default mode network and visual network responsiveness to transcutaneous auricular vagus nerve stimulation predict its variable efficacy in primary insomnia disorder

**DOI:** 10.3389/fneur.2025.1703747

**Published:** 2025-12-16

**Authors:** Xiao Wu, Hua Duan, Yishuang Wang, Shuya She, Yushi Hu, Benxiang He

**Affiliations:** 1 Post-Doctoral Research Station, Affiliated Sport Hospital of Chengdu Sport University, Chengdu, Sichuan, China; 2 Department of Chinese Medicine, Sichuan Provincial People's Hospital, School of Medicine, University of Electronic Science and Technology of China, Chengdu, Sichuan, China; 3 Department of Radiology, Sichuan Provincial People's Hospital, School of Medicine, University of Electronic Science and Technology of China, Chengdu, Sichuan, China; 4 Sichuan Academy of Chinese Medicine Science, Chengdu, Sichuan, China

**Keywords:** transcutaneous auricular vagus nerve stimulation, primary insomnia disorder, heart rate variability, individual efficacy, brain-heart interaction, efficacy prediction

## Abstract

**Backgrounds:**

Transcutaneous auricular vagus nerve stimulation (taVNS) has been proven effective in treating primary insomnia disorder (PID). However, its efficacy exhibits inter-individual variability.

**Objectives:**

This study aimed to investigate the brain functional mechanisms underlying variable efficacy and to identify potential biomarkers that could predict taVNS efficacy.

**Methods:**

We conducted a randomized controlled trial involving 54 PID patients who received real or sham taVNS to assess brain activity and autonomic nervous system (ANS) responses. An additional 46 patients receiving real taVNS were recruited to enlarge the treatment cohort, thereby enhancing the statistical power for biomarker identification.

**Results:**

Post-treatment, the mean amplitude of low-frequency fluctuations (mALFF) of sensorimotor network (SMN), default mode network (DMN), and visual network (VN) in treatment group were significantly increased than those before treatment, and the mALFFs value of the combination of all the differentially significant brain regions (especially DMN + VN) before taVNS were correlated with its efficacy. The heart rate variability indicators “root mean square of successive differences, percentage of adjacent N-N intervals differing by more than 50 ms, and high frequency (HF) during taVNS were significantly greater than pre-treatment. The mALFFs value of DMN and VN before taVNS were correlated with HF during taVNS.

**Conclusion:**

Our findings suggest that taVNS may exerts therapeutic effects in PID through modulating the activities of the DMN (left precuneus and bilateral cuneus), VN (left lingual gyrus, left superior occipital gyrus, left cuneus and bilateral calcarine), and SMN (right precentral, right rolandic operculum, bilateral postcentral gyrus, bilateral paracentral gyrus, bilateral supplementary motor areas and left middle cingulate gyrus), thereby regulating the ANS activity in PID patients. Individual differences in functional responsiveness of the DMN + VN + SMN networks, particularly “DMN (left Precuneus and left cuneus) + VN (left superior occipital and left cuneus)” to ANS modulation during taVNS were correlated with variability in efficacy. Additionally, baseline DMN/VN activity and HF parameters during stimulation demonstrated potential as predictive biomarkers for taVNS efficacy.

**Clinical trial registration:**

This study protocol was registered with the Chinese Clinical Trial Registry (Registration Number: ChiCTR2300076474; accessible at www.chictr.org.cn).

## 1 Introduction

Primary insomnia disorder (PID) is one of the most common mental illnesses worldwide. In addition to an inability to concentrate and a profound sense of fatigue, individuals with PID often experience severe anxiety and depression (1–3). PID severely affects the quality of life and work of the patients, and the resulting high medical expenses and accidents cause considerable social and economic losses (2, 4, 5). Epidemiological data have shown that approximately 1/3 of adults have experienced insomnia, 15% of adults meet the clinical diagnostic criteria for PID, and elderly individuals are the most likely to present with insomnia, with an incidence of 30–50% (2, 6).

Current treatments for PID are based on sedative-hypnotic drugs and cognitive behavioral therapy for insomnia (CBT-I) (7, 8). However, drugs can produce side effects, such as delirium, endocrine disorders, and anterograde memory impairment, which can affect patients’ functioning during the day (9, 10). Long-term use of sleeping pills can even lead to drug dependence and increase the risk of neurological diseases, such as neuritis and Alzheimer’s disease (9, 11). CBT-I has limited efficacy for PID during acute attacks, has poor long-term efficacy, involves a long treatment cycle, and is expensive (12, 13). Therefore, there is an urgent clinical need for treatments that can be used for extended periods, that induce few side effects, and that are convenient to use.

Transcutaneous auricular vagus nerve stimulation (taVNS) is known to be effective in treating PID (14, 15). The auricular vagus nerve branches are distributed across the auricular cavity and the posterior wall of the external auditory canal—the only superficial distribution areas of the vagus nerve on the body, where the skin is thin and sensitive to external stimuli, thus making them ideal areas for noninvasive vagus nerve stimulation (16, 17).

However, the efficacy of taVNS in treating insomnia varies considerably across individuals, which is also a primary concern that hinders its clinical promotion (18–20). Even in diseases approved by the American Food and Drug Administration for taVNS treatment, such as epilepsy and major depression, the effective rate maintain a certain degree of fluctuation (21–23). Therefore, exploring the reasons for this variable efficacy and identifying biomarkers that can predict the efficacy of taVNS is crucial for achieving individualized treatment and saving medical costs. However, few studies have concentrated on the individual differences in taVNS efficacy currently.

Previous research has shown that taVNS could modulate the activity of the brain and the autonomic nervous system (ANS) (24), both of which can be assessed in most hospital settings. Functional magnetic resonance imaging (fMRI), based on blood oxygenation level-dependent (BOLD) signals, has become an important tool for studying brain function mechanisms in the treatment of neurological diseases with various therapies (25, 26). Using fMRI, the changes in the brain regions and brain network activities of PID patients before and after taVNS treatment can be clearly observed (27, 28). The mean amplitude of low-frequency fluctuation (mALFF) is an important parameter derived from fMRI for studying the activity of individual brain regions and their correlations. Many studies have used mALFF to explore the central mechanisms underlying the action of physical therapy in the treatment of neurological diseases and as an objective and stable imaging marker for predicting efficacy (29, 30). Additionally, heart rate variability (HRV) is the most objective and accepted indicator for assessing the state of the human ANS (vagal tone) and for observing the effects of taVNS on vagal tone (31–33).

Accordingly, we employed mALFF values to assess the effects of taVNS on human cortical activity and used HRV to evaluate its effects on the autonomic nervous system (ANS). The efficacy of taVNS was evaluated using the two most widely used scales for assessing insomnia severity, the Insomnia Severity Index (ISI) and the Pittsburgh Sleep Quality Index (PSQI) (34–36). Then we analyzed the correlations among these factors to explore the brain functional mechanisms that may cause the variable efficacy of taVNS and to identify potential biomarkers for predicting taVNS efficacy.

## 2 Methods

### 2.1 Patients

This study protocol was approved by the Ethics Committee of the Sichuan Academy of Medical Sciences & Sichuan Provincial Hospital (Approval Number: 2023–391) and was registered with the Chinese Clinical Trial Registry (Registration Number: ChiCTR2300076474; accessible at www.chictr.org.cn). All participants provided written informed consent prior to enrollment.

The sample size was estimated based on the formula for the minimum sample size per group, as outlined in the 4th edition (2015) of Medical Statistics edited by Professor Sun Zhenqiu (People’s Medical Publishing House, p. 573). The formula is as follows:


$$n=\frac{{\left(u_{\alpha }+u_{\beta }\right)}^{2}(1+\frac{1}{k})\sigma^{2}}{\delta^{2}}$$

$\delta =|{\bar{x}}_{1}-{\bar{x}}_{2}|$ , $\sigma^{2}$ was the pooled standard deviation of two groups, with the parameter settings of *α* = 0.05, *β* = 0.05, and a two-tailed test. The sample size was calculated with the Pittsburgh Sleep Quality Index (PSQI) as the primary endpoint (37). The minimum required sample size was determined to be 52. This necessitates at least 26 participants per group. To account for an anticipated 5% dropout rate, the adjusted total sample size was calculated as “52 / (1–0.05) = 54.73,” which was rounded up to 55. However, considering the generally high compliance observed among chronic insomnia patients in our clinics, we recruited 27 participants per group.

**Figure 1:**
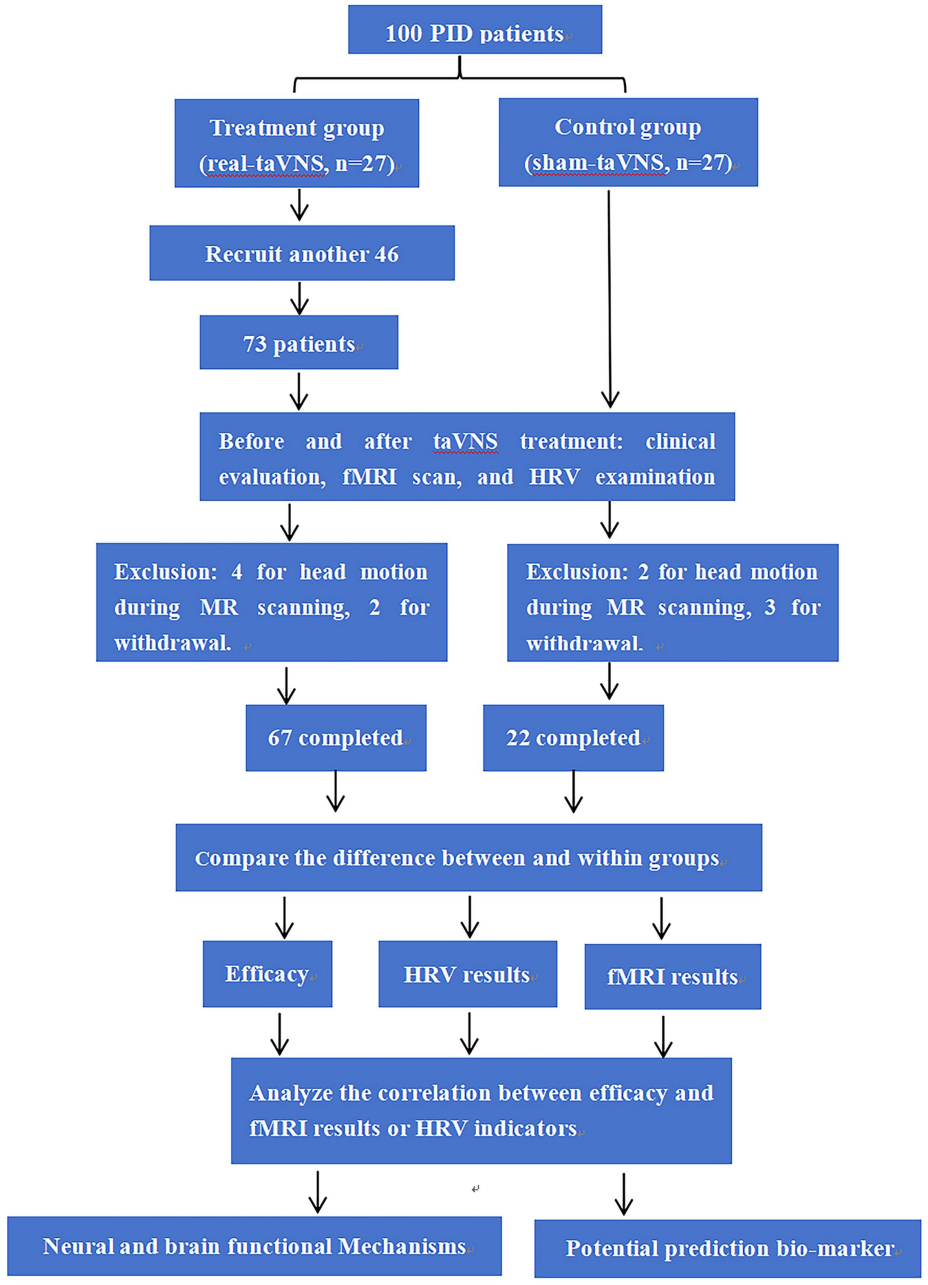
The flowchart of our study.

We conducted a randomized controlled trial (RCT) involving 54 PID patients who received real or sham taVNS to assess brain activity and autonomic nervous system (ANS) responses. An additional 46 patients receiving real taVNS were recruited to enlarge the treatment cohort, thereby enhancing the statistical power for biomarker identification. All patients were enrolled from the outpatient clinic of Department of Chinese Medicine, Sichuan Provincial People’s Hospital. The first 54 PID patients (out of 100) were assigned random numbers generated by the “r function” in the 2020 version of “WPS-Excel” software. Patients 1–27 (the first half) were assigned to the treatment group, and patients 28–54 (the latter half) served as the control group. A total of 67 patients in the treatment group and 22 patients in the control group completed treatment and imaging examinations (Figure 1).

The diagnosis and eligibility of all PID patients were confirmed by two senior physicians from the Department of Chinese Medicine, specializing in sleep disorders. In cases of diagnostic uncertainty, the chief physician was consulted for a final decision. All patients provided written informed consent before enrollment and underwent magnetic resonance imaging (MRI) both before and after the taVNS treatment. Relevant clinical scales were assessed before treatment and within 3 days after its completion.

### 2.2 Inclusion criteria

(1) Age 18–70 years (inclusive); right-handedness; at least one of the three major symptoms of difficulty falling asleep, insufficient sleep time, and difficulty maintaining sleep lasting for more than 3 months and occurring 3 times a week, satisfying the clinical diagnostic criteria of PID in *The Diagnostic and Statistical Manual of Mental Disorders, 5th Edition* by the American Psychiatric Association; PID diagnosed via polysomnography; and the presence of daytime fatigue, an inability to concentrate, drowsiness, emotional instability, and other conditions that affect daytime functioning (4).
(2) PSQI > 7 points, ISI > 14 points, 24-item Hamilton Depression rating scale (HAM-D) score < 20 points, Hamilton Anxiety rating scale (HAM-A) score < 21 points, self-rating anxiety scale (SAS) standard score < 69, and self-rating depression scale (SDS) standard score < 72 points.
(3) Difficulty falling asleep that cannot be explained simply by the sleep environment (such as noise, light, and comfort) and inability to explain the cause of the patient’s insomnia by another disease in the absence of other neurological diseases.
(4) No use of any sedative hypnotics in the past month.
(5) Willingness to participate in this study, a junior high school education level or above, and ability to cooperate with all examinations.

### 2.3 Exclusion criteria

(1) Noncompliance with any of the above inclusion criteria.
(2) Age < 18 years and > 70 years (not inclusive).
(3) A history of head trauma or other neurological diseases other objective environmental causes that could explain the insomnia.
(4) Serious primary diseases, such as tumors of the digestive system, hematopoietic system or endocrine system, and contraindications to vagus nerve stimulation, such as heart failure and hyperthyroidism.
(5) PSQI < 7 points, ISI < 8 points, 24-item HAMD score ≥ 20 points, HAMA score ≥ 21 points, SAS standard score ≥ 69 points, and SDS standard score ≥ 72 points.
(6) A history of drug abuse or alcohol abuse.
(7) Skin allergies, injury or inflammation at the concha cavity and external auricle; pregnancy or planning for pregnancy; and lactation.
(8) Previous participation in other clinical trials in the past 6 months; previous use of anticholinergic drugs, sedative hypnotics, and hormonal drugs within 1 month; or current irregular use of drugs that inhibit the cerebral cortex or nervous system.

### 2.4 Interventions

**Figure 2:**
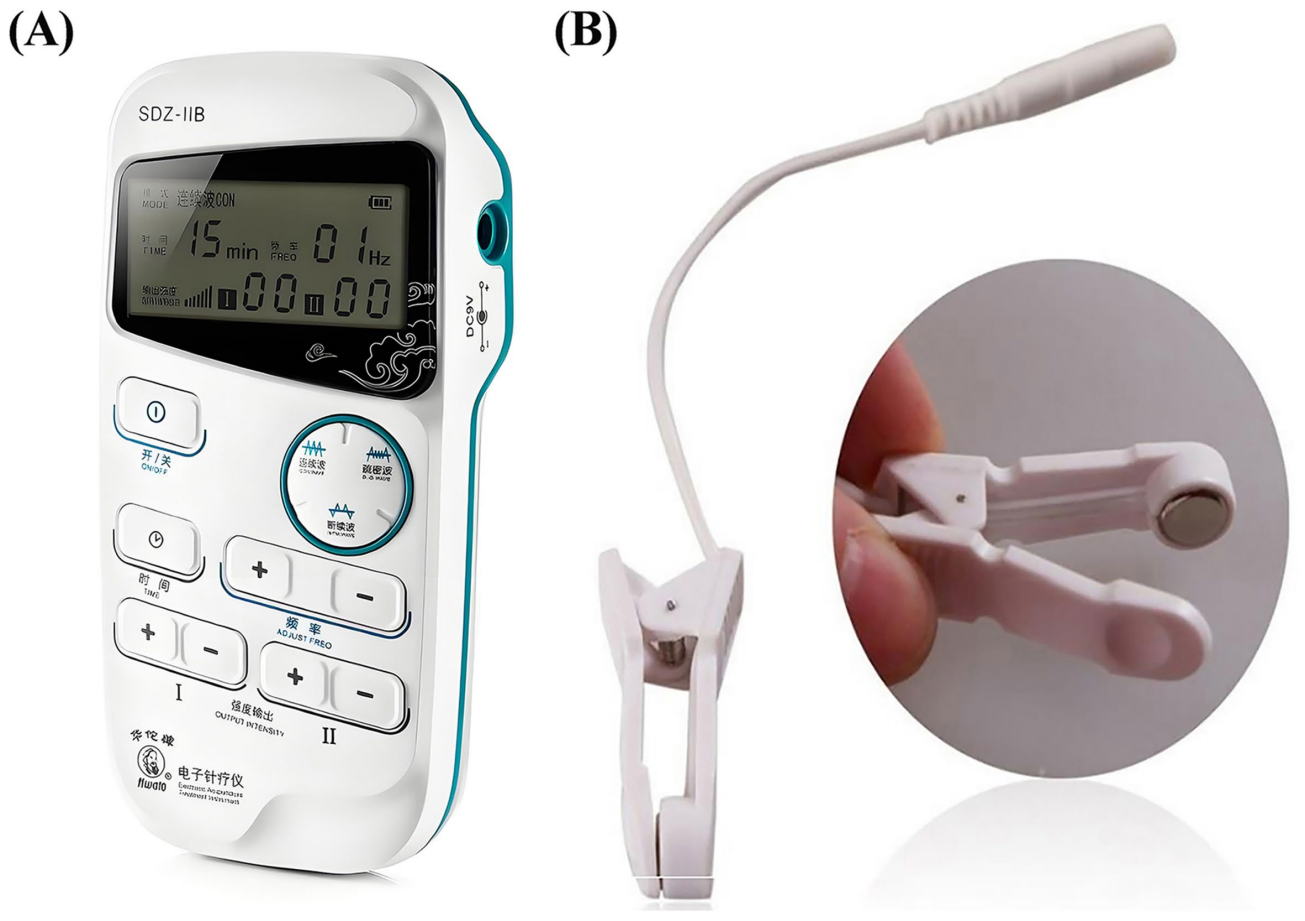
The electroacupuncture device and ear clips of taVNS. **(A)** The electroacupuncture device. **(B)** The ear clips.

We used a Hwato electroacupuncture device (Hwato, SDZ-IIB, Suzhou, China) (Figure 2) and currently widely used taVNS interventions and parameters (18, 37). Patients self-reported their daily sleep/wake schedules and treatment adherence in a study diary. To promote compliance, research staff performed telephone follow-ups with each patient every third day.

#### 2.4.1 Interventions for the treatment group (real taVNS group)

**Figure 3:**
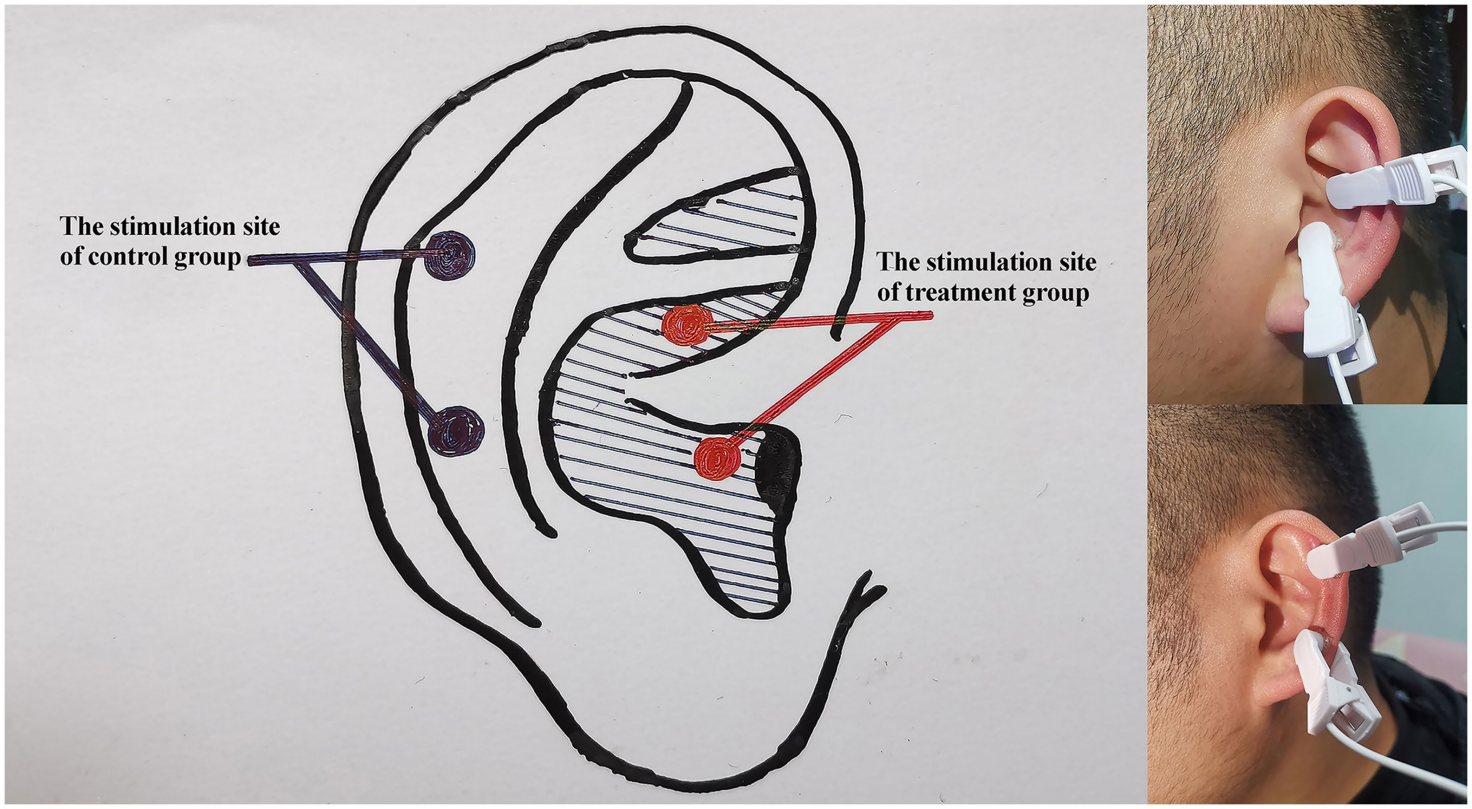
Schematic of the treatment sites. The shaded area represents the distribution area of the vagus nerve; red represents the stimulation sites in the treatment group, and black represents the stimulation sites in the control group.

Stimulation sites: The taVNS point was located in the concha cavity region of the left pinna, where there are abundant vagus nerve branches (Figure 3). Interventions and stimulation parameters: Patients were placed in the sitting or supine position. After the stimulation sites were disinfected according to standard practice, ear clips were attached to the stimulation sites in the concha cavity (18, 37).

Stimulation parameters: (1) waveform: longitudinal waves; (2) wave-width: 200 ms; (3) frequency: 20 Hz; and (4) stimulation intensity: adjusted by the patient according to his or her own tolerance; a visual analog scale (VAS) score of 3 (12–17 mA) was generally required. Each treatment lasted 30 min and performed twice daily—once at noon and once before sleep—for at least 5 days a week for 4 weeks (18, 37, 38).

#### 2.4.2 Interventions for the control group (sham taVNS group)

Stimulation site: The taVNS point was located outside the vagus nerve distribution area of the external ear (Figure 3). Patients were placed in a sitting or supine position. After disinfection of the stimulation site according to standard practice, the ear clips were attached to the stimulation site (39).

Stimulation parameters: It’s the same as those in the treatment group.

### 2.5 Clinical evaluation

The severity of PID in the patients was evaluated via the PSQI and ISI before and after treatment.

### 2.6 MRI data acquisition

All fMRI examinations were conducted on a 3.0T Philips Elition X MR scanner (Amsterdam, Netherlands) with a 32-channel birdcage head coil. The subjects were instructed to stay awake, remain motionless, and think about nothing. Each scanning session lasted approximately 20 min. The MRI sequences included three-dimensional (3D) high-resolution anatomical imaging (Magnetization Prepared-RApid Gradient Echo, MPRAGE), an 8-min resting-state functional MRI scan before treatment and another 4 weeks after taVNS treatment.

Functional images were obtained with the following parameters: field of view (FOV): 240 mm × 240 mm × 142 mm, voxel size: 3.75 mm × 3.75 mm × 3.5 mm, matrix: 64 × 61 × 38 slices, repetition time (TR): 2000 ms, echo time (TE): 30 ms, dynamic scans: 240, slices: 38, slice gap: 0.25 mm, slice scan order: interleaved, patient position: head first, patient orientation: supine, frequency direction: anterior/posterior (A/P), flip angle: 90°, SPEEDER PE: 1, fat suppression: spectral presaturation with inversion recovery (SPIR), slice orientation: sagittal, and scan time: 8:06 s.

The parameters of the (3D) high-resolution full-brain structural image (T1) were as follows: FOV: 256 mm × 240 mm × 224 mm, voxel size: 0.8 × 0.8 × 0.8 mm, matrix: 320 × 300 × 280 slices, TR: 9.9 ms, TE: 4.6 ms, slices: 280, slice gap: 0 mm, head position: first, fat suppression: no, patient orientation: supine, frequency direction: A/P, flip angle: 8°, slice orientation: sagittal, scan time: 5:14 s, PNS mode: moderate.

### 2.7 Resting state functional imaging data processing

The resting-state fMRI data preprocessing was performed using RESTplusV1.28 (developed by Prof. Yufeng Zang’s team at Hangzhou Normal University Affiliated Hospital) and SPM V12.0 (developed by University College London) based on MATLAB (The Math Works, Natick, MA, United States) (40, 41). For each subject, the data were processed by the following eight steps: (1) Convert the functional magnetic resonance imaging (fMRI) data from DICOM format to NIFTI format. (2) the images corresponding to the first 10 time points were removed for magnetization equilibrium, and the other 230 volumes were used for further analysis; (3) slice-timing correction was conducted to adjust the acquisition time delay between slices; (4) realignment of head motion: images demonstrating translations in the x, y, or z direction exceeding 3 mm and rotations exceeding 3° were eliminated. (5) The functional data were subsequently registered to high-definition T1 structural phase images and normalized with the Diffeomorphic Anatomical Registration Through Exponential Lie (DARTEL) method. Then, the normalized functional images were resampled to Montreal Neurological Institute (MNI) space, resampled to a 3-mm isotropic resolution; (6) smooth the resampled data with a Gaussian kernel at a full width at half maximum of 6 × 6 × 6 mm. (7) The 24-head motion parameters, white matter signals, and cerebrospinal fluid signals were linearly regressed via a generator, and linear trends were removed from the fMRI data. (8) The ALFF values obtained by squaring the power spectrum of the signal from 0.01 Hz to 0.08 Hz. The mALFF was subsequently obtained by dividing the ALFF values by their mean.

### 2.8 Statistical analysis of fMRI data

A paired-sample *t*-test was used to compare the mALFF values within each group before and after treatment, with the mean head-motion value (mean FD Jenkinson) as covariate variables. A threshold of voxel-wise p uncorrected and a cluster-level p corrected by family wise error were applied for multiple-comparison corrections. If voxel wise *p* < 0.05 and cluster-level *p* < 0.05, the difference was statistically significant.

Besides this, we, respectively, extracted the average T values of significantly altered clusters (before and after treatment). Then, the differences of the mALFF values were compared using a paired-sample *t*-test, respectively, and *p* < 0.05 was considered to be statistically significant.

### 2.9 Heart rate variability data acquisition

An echocardiography (ECG) device (ECG-3303B, Guangdong Medical; registration number: 20182070863) and an information management system from Guangzhou Sanrui Electronic Technology Co., Ltd. were used to record and analyze the 5-min ECG and HRV data of the patients before and after taVNS treatment.

### 2.10 Clinical outcomes and statistical analysis

Data entry and statistical analysis were performed with SPSS for Windows version 18.0 (SPSS, Inc., Chicago, IL, United States). Continuous variables are expressed as the mean (standard deviation, SD), and qualitative data are expressed as proportions and rates. The Shapiro–Wilk test was used to assess the normality of the data distributions, and the Levene test was used to assess the homogeneity of variance. Normally distributed variables with assumed homogeneity of variance were compared with the independent samples t test, and non-normally distributed variables or those without homogeneity of variance were compared with the Mann–Whitney *U* test. Categorical variables are expressed as numbers, and differences between groups were assessed with Pearson’s *χ*^2^ test or Fisher’s exact test. Correlations between normally distributed variables were assessed with Pearson correlation analysis, and those between non-normally distributed variables were assessed with Spearman sequential correlation analysis. A two-tailed *p*-value less than 0.05 was considered statistically significant. As the correlation analysis was conducted among three categories of indicators (clinical, imaging, and HRV), we applied a Bonferroni-corrected significance level of *p* < 0.015 (0.05/3) to declare a statistically significant correlation.

## 3 Results

### 3.1 Participants and baseline characteristics

Overall, 73 patients received real taVNS, and 27 patients received sham taVNS. By the end of the study, 67 patients in the treatment group and 22 patients in the control group had completed the treatment and MRI examinations, and their data were used for subsequent analyses (Figure 1). The demographic data of these 89 patients are shown in Table 1.

**Table 1:** Demographic and baseline data of the patients.

Characteristic		Treatment group mean (*n* = 67)	Control group (*n* = 22)	*t*/*Z*/*χ*^2^	*P*-value
Age		45.40(13.73)	46.36(13.41)	−0.362	0.718
BMI		21.89(2.76)	21.89(2.59)	−0.004	0.997
Insomnia duration		69.83(70.38)	70.55(80.70)	−0.153	0.878
SBP (mmHg)		121.76(13.28)	121.18(12.44)	0.18	0.857
DBP (mmHg)		75.12(10.27)	74.18(8.47)	0.387	0.70
Sex	Male	20(29.9%)	7(31.8%)	0.03	0.862
	Female	47(70.1%)	15(68.2%)		
Smoking	Yes	1(1.5%)	2(9.1%)	0.00	1.00
	No	66(98.5%)	20(90.9%)		
Drinking	Yes	3(4.5%)	1(4.5%)	0.00	1.00
	No	64(95.5%)	21(95.5%)		
Hypertension	Yes	3(4.5%)	0(0%)		0.315
	No	64(95.5%)	22(100%)		
Allergy	Yes	12(17.9%)	1(4.5%)	1.421	0.233
	No	55(82.1%)	21(95.5%)		

BMI, body mass index; SD, standard deviation; SBP, systolic blood pressure; DBP, diastolic blood pressure.

### 3.2 Clinical outcomes

**Figure 4:**
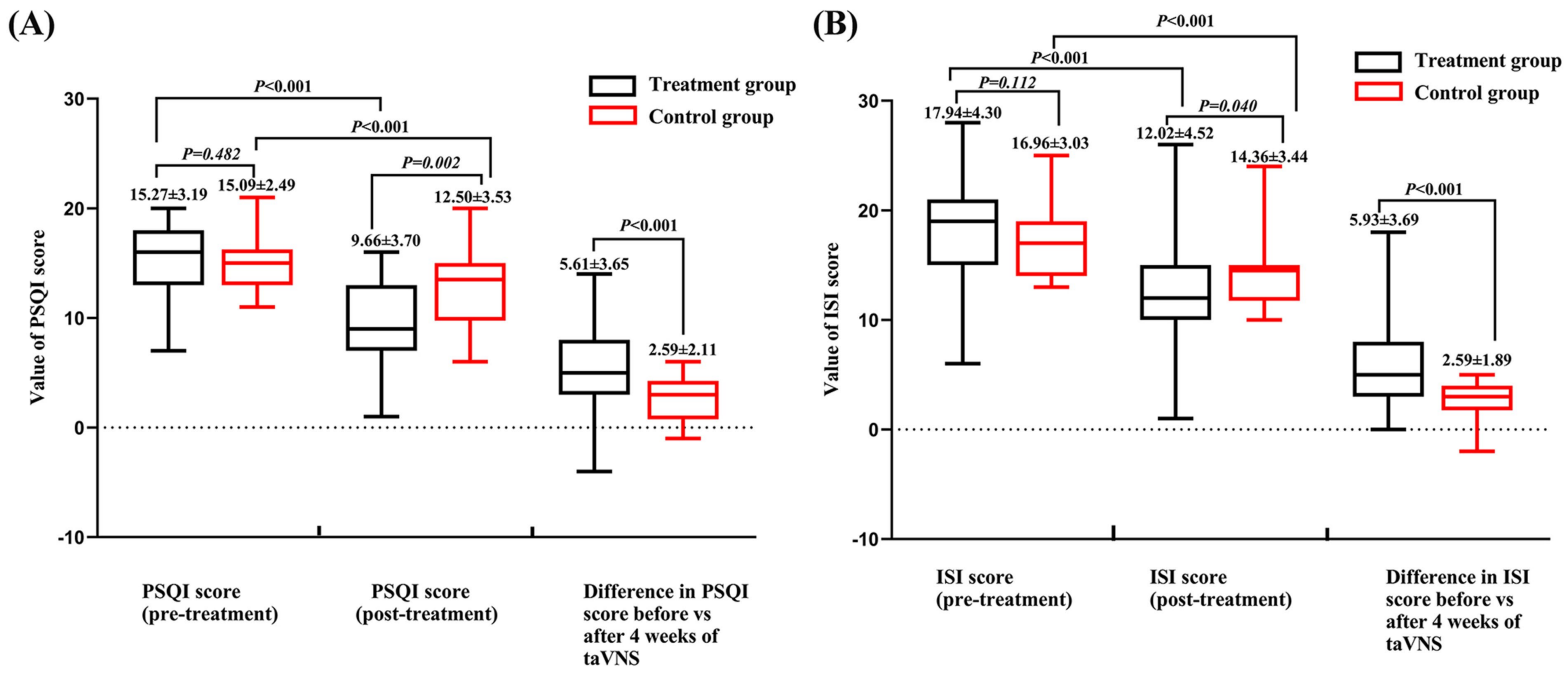
PSQI and ISI score of the treatment and control group before and after treatment. **(A)** PSQI score. **(B)** ISI score.

The clinical outcome results showed: Both treatment group and control group got improvement on PSQI and ISI score after taVNS treatment. While the treatment group showed greater improvement than control group (Figure 4 and Tables 2, 3).

**Table 2:** Pittsburgh Sleep Quality Index (PSQI) of the treatment and control groups before and after treatment.

Item	Treatment group	Control group	*t/Z*	*P*
Pre-treatment	15.27 ± 3.19*	15.09 ± 2.49	−0.703	0.482
Post-treatment	9.66 ± 3.70	12.50 ± 3.53	−3.163	0.002
*t/Z*	−6.865	−5.765		
*P*	0.000	0.000		
Difference of PSQI score values (pre and post treatment)	5.61 ± 3.65	2.59 ± 2.11	3.680	0.000

*Non-normal distribution.

**Table 3:** Insomnia Severity Index (ISI) of the treatment and control groups before and after treatment.

Item	Treatment group	Control group	*t/Z*	*P*
Pre-treatment	17.94 ± 4.30*	16.96 ± 3.03	−1.591	0.112
Post-treatment	12.02 ± 4.52	14.36 ± 3.44*	−2.058	0.040
*t/Z*	−7.017	−3.746		
*P*	0.000	0.000		
Difference of ISI score values (pre and post treatment)	5.93 ± 3.69*	2.59 ± 1.89	−3.885	0.000

*Non-normal distribution.

### 3.3 HRV results

**Figure 5:**
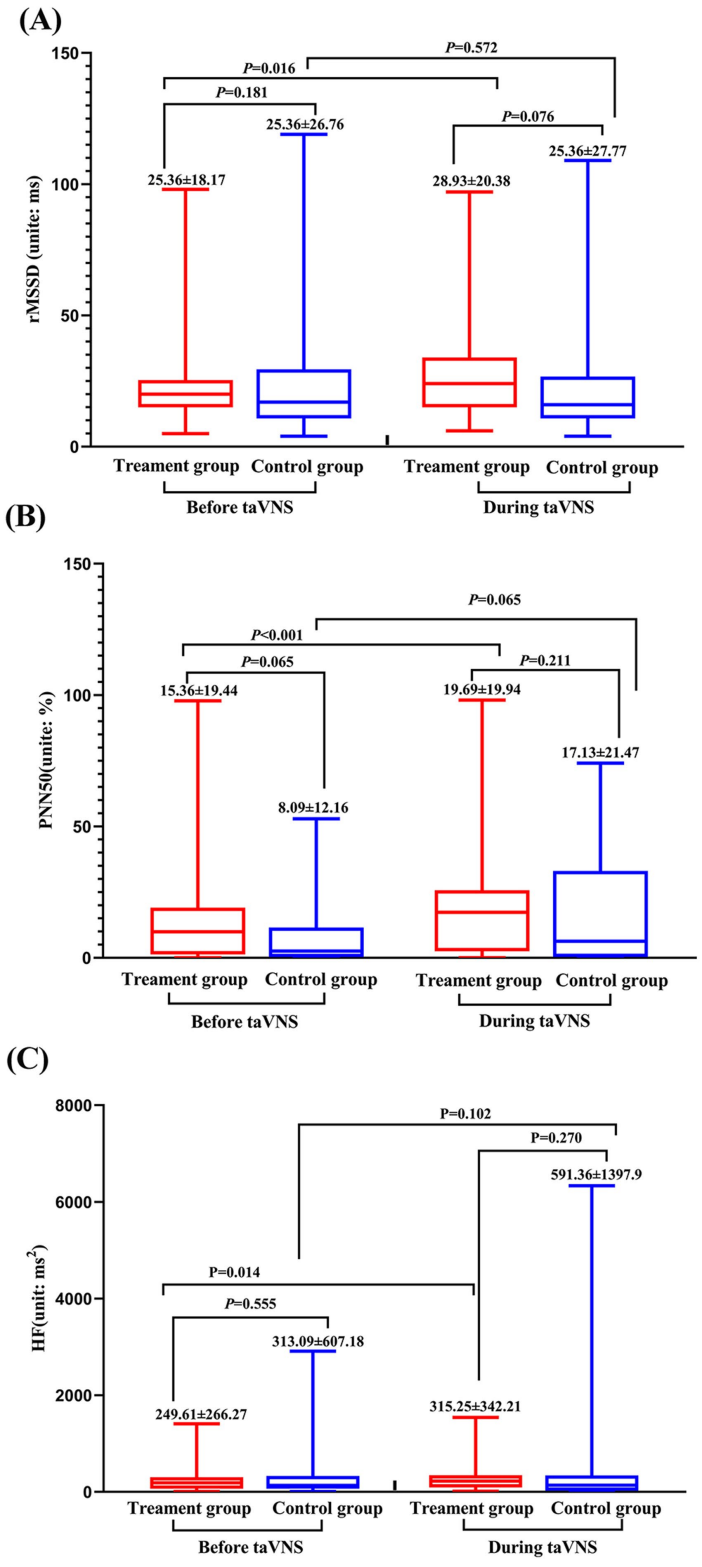
Heart rate variability parameters of treatment and control group before and during continuous taVNS. **(A)** rMSSD; **(B)** PNN50; **(C)** HF.

The comparison of HRV results showed: Despite there is no significant difference between the treatment group and the control group, but the root mean square of successive differences (rMSSD), high frequency (HF) and percentage of differences exceeding 50 ms between adjacent normal RR intervals (PNN50) of treatment group were greater during continuous taVNS than the un-stimulated state (*p* > 0.05). In contrast, only PNN50 showed alteration during taVNS in control group (*p* < 0.05) (Figure 5).

### 3.4 fMRI results

#### 3.4.1 Treatment group

**Figure 6:**
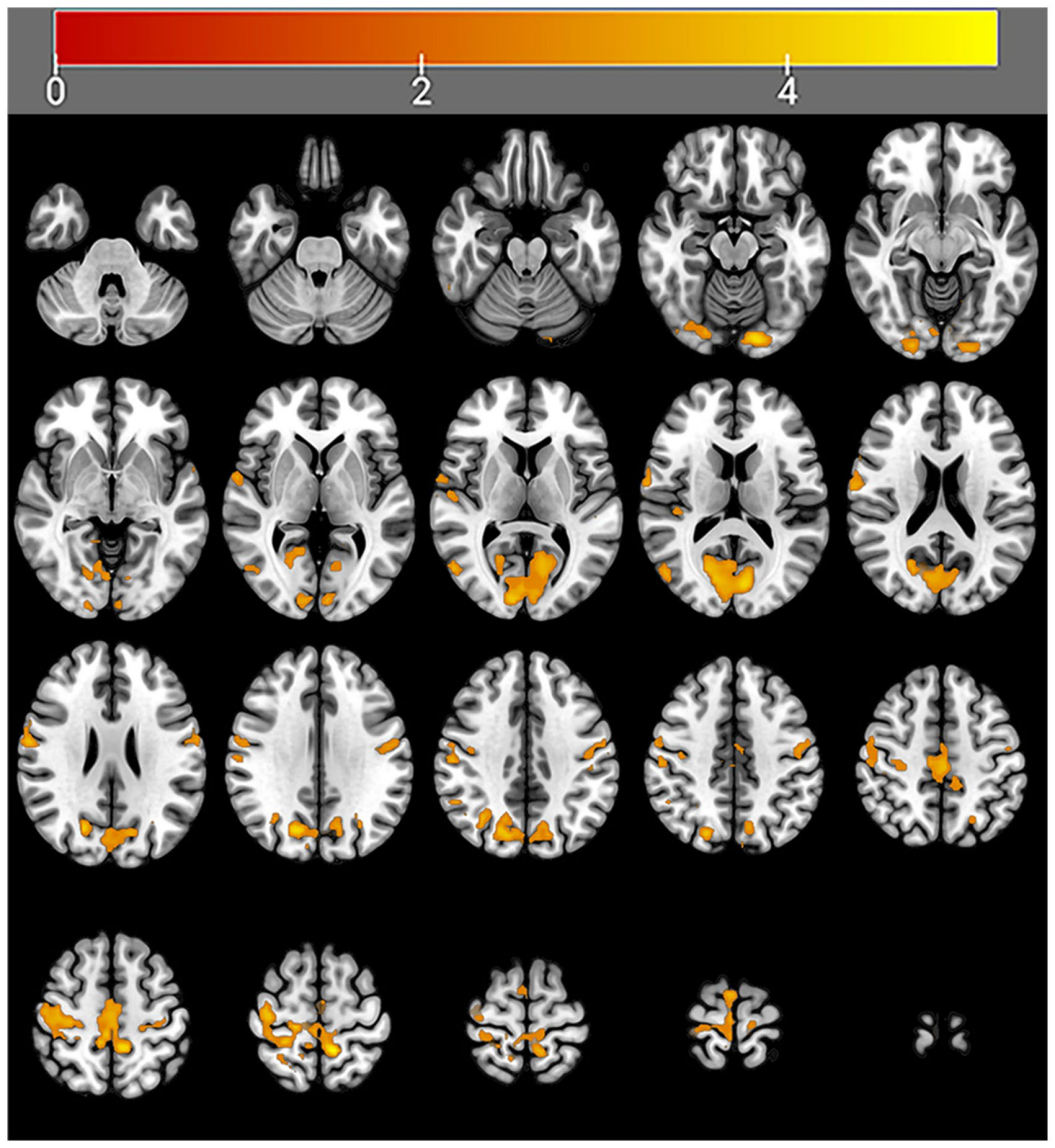
Regions with increased brain activity in the treatment group after 4 weeks of taVNS treatment. Color intensity (yellow) corresponds to the magnitude of the increase in activity in these brain regions.

**Figure 7:**
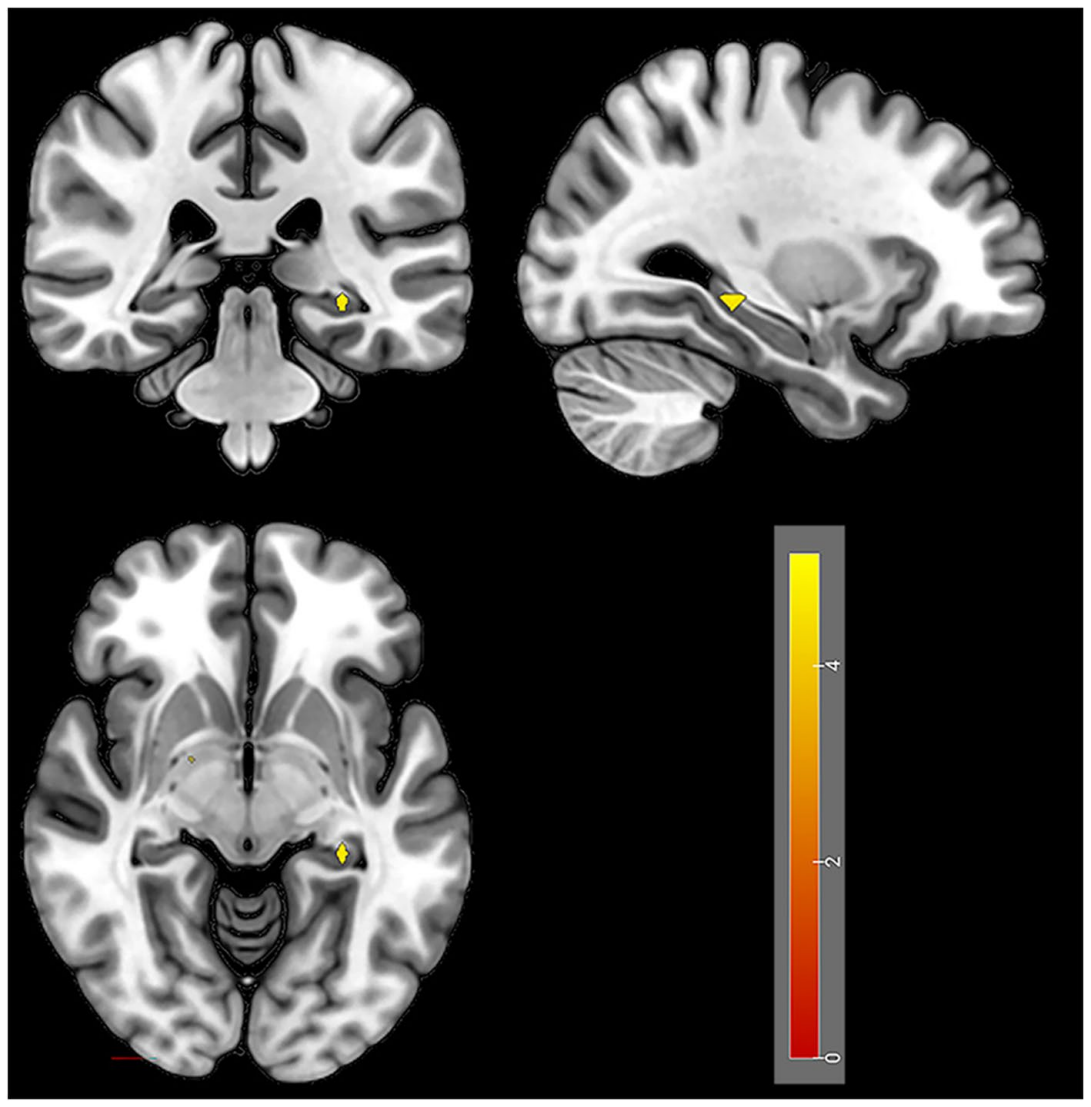
Regions with decreased brain activity in the treatment group after 4 weeks of taVNS treatment. Color intensity (yellow) corresponds to the magnitude of the decrease in activity in these brain regions.

After 4 weeks of taVNS treatment, the mALFF value in the sensorimotor network (SMN) brain regions (Precentral_R, Rolandic operculum_R, Postcentral gyrus_Bi, Paracentral_Bi, Supplementary motor areas_Bi and Middle cingulate gyrus_L), default mode network (DMN) brain regions (Precentral_R, Rolandic operculum_R, Postcentral gyrus_Bi, Paracentral_Bi, Supplementary motor areas_Bi and Middle cingulate gyrus_L), and visual network (VN) brain regions (Lingual gyrus_L, Superior occipital gyrus_L, Cuneus_L and Calcarine_Bi) of the treatment group was significantly greater than that before the treatment (Table 4 and Figure 6). In contrast, the mALFF in the right hippocampus decreased post-treatment (Table 4 and Figure 7).

**Table 4:** Regions showing significantly increased mALFF values after taVNS treatment compared with “before treatment,” controlling for age as a covariate (voxelwise, *P* < 0.05, uncorrected; clusterwise, *P* < 0.05, FDR corrected).

Group	Contrast	Cluster	Voxel-size	Brain region	Peak *T*-value	MNI coordinates		
						*X*	*Y*	*Z*
Treatment group	Post>Pre	Cluster 1	53	Lingual gyrus_L	4.98	−15	−87	−15
		Cluster 2	469	Calcarine_Bi	5.16	−9	−81	12
				Cuneus_Bi				
				Precuneus_R				
		Cluster 3	96	Rolandic operculum_R	4.57	66	−3	15
		Cluster 4	49	Precuneus_L	4.02	−15	−66	36
				Cuneus_L				
				Superior occipital_L				
		Cluster 5	227	Postcentral_Bi	4.48	39	−15	60
				Precentral_R				
		Cluster 6	225	Paracentral_Bi	4.86	−12	−45	60
				SMA_Bi				
				Middle cingulum_L				
	Post<Pre	Cluster 1	6	Hippocampus_R	5.12	39	−30	−9
Control group	Post>Pre		No brain region above the threshold.					
	Post <Pre		No brain region above the threshold.					

mALFF, mean amplitude of low-frequency fluctuation (mALFF); taVNS, transcutaneous auricular vagus nerve stimulation; SMA, supplementary motor area.

#### 3.4.2 Control group

In the sham taVNS group, no significant changes in brain function were observed before or after treatment.

### 3.5 The results of correlation analysis

**Figure 8:**
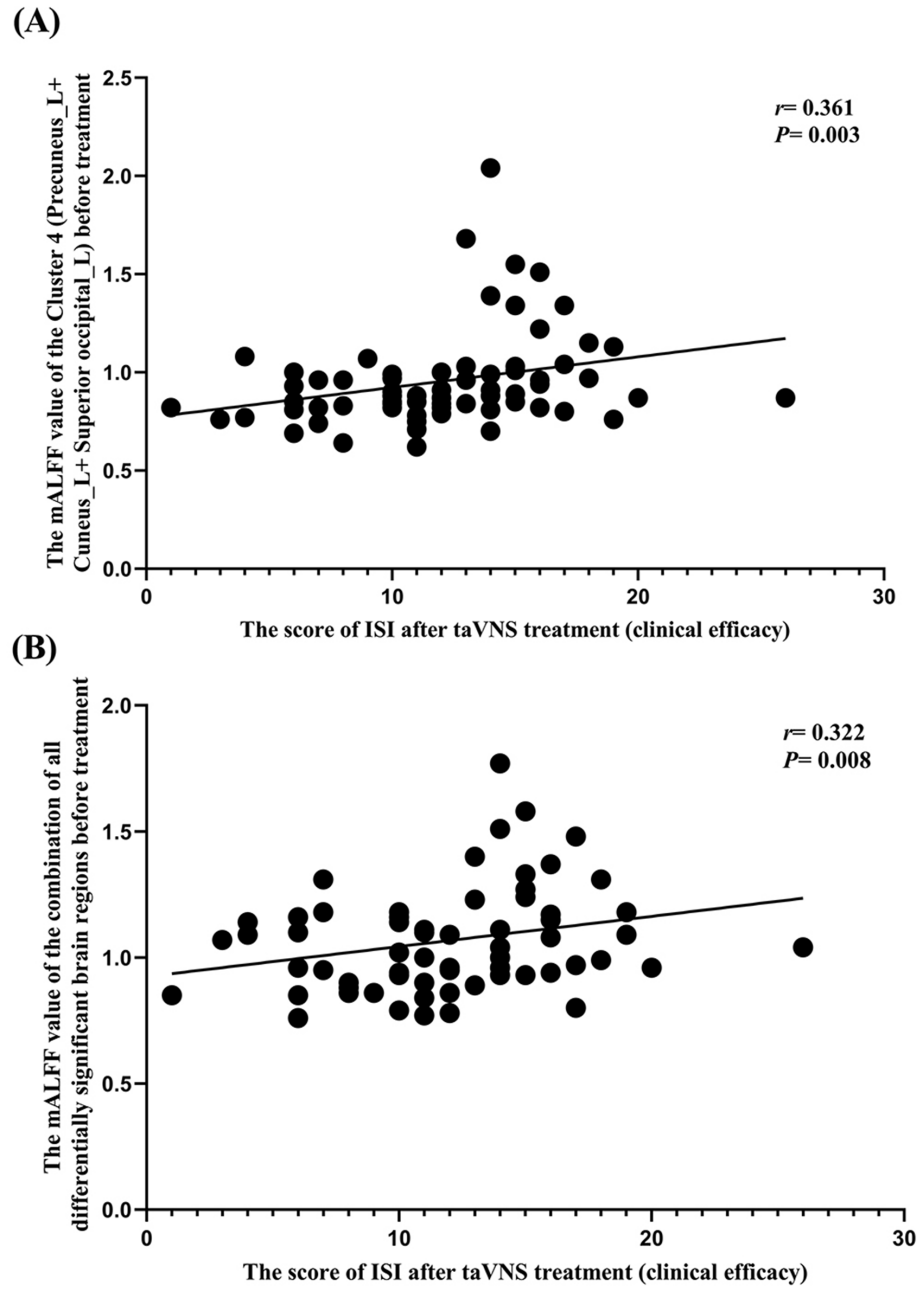
The correlation analysis results between clinical efficacy and the mALFF value before taVNS treatment. **(A)** The correlation results between the ISI score after treatment and the mALFF values of the combination of all the differentially significant brain regions before treatment (*r* = 0.322, *p* = 0.008). **(B)** The correlation results between the ISI score after treatment and the mALFF values of Cluster 4 (Precuneus_L + Cuneus_L + Superior occipital_L) before taVNS treatment (*r* = 0.361, *p* = 0.003). Note: Precuneus_L + Cuneus_L” belong to DMN, and “Superior occipital_L + Cuneus_L” belong to VN.

**Figure 9:**
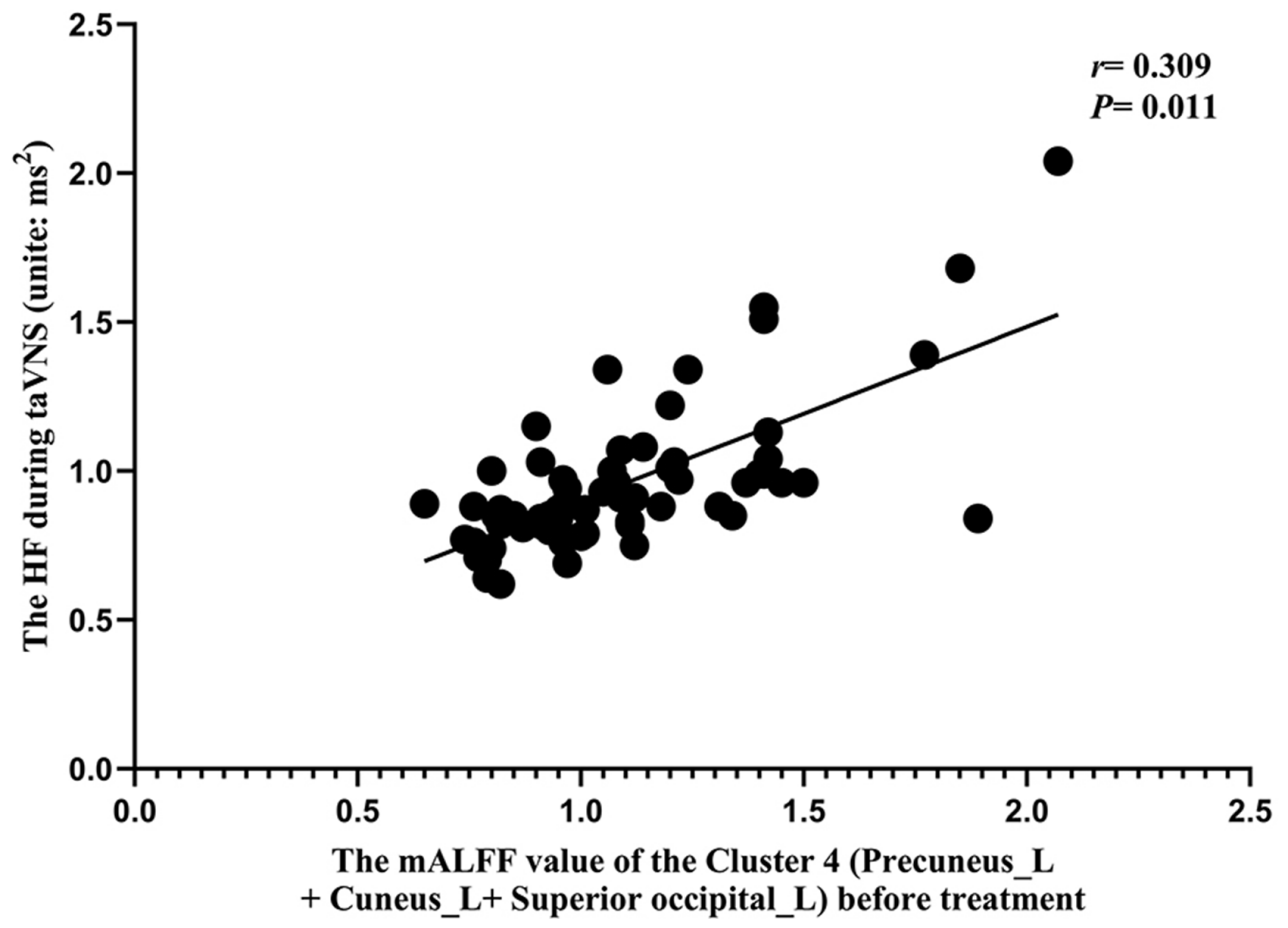
The correlation analysis results between HRV indicators during taVNS and mALFF values before taVNS treatment. The correlation results between HF during taVNS and the mALFF values of Cluster 4 (Precuneus_L + Cuneus_L + Superior occipital_L) before taVNS treatment (*r* = 0.309, *p* = 0.011). Note: “Precuneus_L + Cuneus_L” belong to DMN, and “Superior occipital_L + Cuneus_L” belong to VN.

The results of the correlation analysis showed that the mALFF value before taVNS treatment was associated with the efficacy of taVNS and the HRV indicator during taVNS treatment (*p* < 0.015) (Figures 8, 9). The results of the correlation analysis showed that the pre-treatment mALFF value of cluster 4 and the combination of all differentially significant brain regions was significantly associated with both the efficacy of taVNS and the HRV indicator during taVNS treatment (*p* < 0.015; Figures 8, 9).

## 4 Discussion

The following results were obtained in this study: (1) Post-treatment, the mean amplitude of low-frequency fluctuations (mALFF) of the sensorimotor network (SMN), default mode network (DMN), and visual network (VN) in the treatment group were significantly increased compared to those before treatment. Furthermore, the combined mALFF value of all brain regions showing significant differences (especially DMN and VN) before taVNS was correlated with its efficacy. (2) The HRV indicators (rMSSD, pNN50, HF) during taVNS were significantly greater than the pre-treatment values. (3) The mALFF value of the DMN and VN before taVNS was correlated with the HF during taVNS.

Previous studies have demonstrated the efficacy of taVNS in treating PID (15, 38). For example, Zhang et al. (15) revealed that taVNS can significantly improve sleep quality and mental health, while also alleviating fatigue in PID patients. Additionally, Szulczewski et al. (42) reported that taVNS can improve PID symptoms and alter heart rate variability (HRV). They further suggested that the efficacy of taVNS could be enhanced when combined with slow-breathing exercises to increase vagus nerve stimulation (42). Our study demonstrated that Pittsburgh Sleep Quality Index (PSQI) and Insomnia Severity Index (ISI) scores improved in both the treatment and control groups after the intervention. However, the improvement observed in the treatment group was significantly greater than that in the control group. Since some studies have shown that the development of insomnia is closely related to psychological well-being, the symptomatic improvement in the control group may be attributed to a placebo effect (15, 43, 44).

In recent years, researchers have explored the mechanism of taVNS in treating PID from multiple perspectives. Physiologically, Ma et al. (45) and Tian et al. (46) reported that PID patients often exhibit disorders in ANS and elevated levels of inflammatory factors in the nervous system. They proposed that since taVNS can mitigate both of these conditions, this may represent a key mechanism of its therapeutic action (45, 46). From a biochemical perspective, Manta et al. (47) suggested that long-term vagus nerve stimulation could change the distribution of neurotransmitters in rat monoaminergic neuronal brain regions, which are brain areas closely involved in the development of PID. From the perspective of imaging studies, Zhang et al. (48) found that PID patients frequently show DMN dysfunction, and proposed that taVNS could treat insomnia through the regulation of this network. Zhao et al. (37) suggested that the thalamus is an important brain region in the regulation of sleep and that taVNS may play an important role in PID by regulating thalamic activity. These studies all demonstrated the possible mechanism of taVNS in treating PID from different perspectives.

Current studies on the mechanisms of taVNS have mainly focused on brain function. However, most of them have only compared its effects between PID patients and healthy individuals, lacking direct comparisons of the effects of real versus sham taVNS on brain activity or HRV in PID patients. Therefore, our study compared real and sham stimulation to examine their differential effects on brain function (using mALFFs) and HRV in PID patients. This approach aims to clarify the underlying neural mechanisms of taVNS and to identify the specific brain regions and HRV indicators modulated by this treatment.

Our study revealed that the mALFFs value of SMN (right precentral gyrus, right rolandic operculum, bilateral postcentral gyrus, bilateral paracentral, bilateral supplementary motor areas and left middle cingulate gyrus), DMN (left precuneus and bilateral cuneus), and VN (left lingual gyrus, left superior occipital gyrus and bilateral calcarine) were significantly greater than those before the treatment, while the activity of hippocampal was significantly lower than that before treatment in the treatment group; however, no significant change was observed in the brain functional activities of the control group after treatment.

The DMN is widely implicated in the pathophysiology of insomnia. For instance, Schiel et al. (49) demonstrated that patients with PID exhibit significantly increased activity within the DMN, a pattern that correlates with reports of intrusive thoughts and an inability to control mental processes at bedtime. Hildebrand L et al. also reported that in PID patients, changes in the DMN were significantly positively correlated with the PSQI (50). Zhang et al. (48) reported that the medial prefrontal cortex (mPFC), a core hub of the DMN, is crucial for sleep maintenance. Their study found that taVNS can modulate the functional connectivity (FC) between the mPFC and the occipital lobe in PID patients. Importantly, this enhanced FC was positively correlated with improvements in both sleep duration and sleep quality, suggesting it may be one mechanism through which taVNS treats PID (48). Our study revealed that after taVNS treatment, the mALFFs in the DMN brain regions (left precuneus, left cuneus, and bilateral temporal lobes) of PID patients were significantly increased. This finding is consistent with previous studies and indicates that taVNS may exert a therapeutic effect in PID by regulating DMN activity.

The SMN is primarily responsible for motor control, hearing, and the processing of somatosensory and external stimuli. In PID patients, functional abnormalities of the SMN often manifest as hypersensitivity to sound and various physical stimuli (18, 51). Hsu et al. (52) and Ma et al. (53) demonstrated that the SMN plays a critical role in sleep quality and that alterations in SMN activity are particularly pronounced in patients with sleep deprivation and fragmentation. While, the function of the SMN can change after professional treatment. For example, Ning et al. (51) reported that acupuncture significantly reduced the functional connectivity (FC) between the SMN and both the dorsal attention network and the frontoparietal network in PID patients. This reduction may explain how acupuncture exerts its therapeutic effect on insomnia (51). Badran et al. (54) reported that continuous taVNS treatment activated the bilateral cerebellum and superior frontal gyrus within the SMN, whereas no significant changes were observed in these regions under sham stimulation. Supporting this, our study found that after taVNS treatment, the activity of key SMN regions (precentral gyrus, bilateral postcentral gyrus, and bilateral supplementary motor areas) in PID patients was significantly greater than pre-treatment levels, indicating that the SMN may be a key target of taVNS for treating PID.

The VN is primarily responsible for vision and visual processing. In patients with PID, VN abnormalities often manifest as hypersensitivity to light and difficulty initiating sleep (55). Dai et al. (56) reported that PID patients exhibit functional abnormalities in the VN and altered FC between the VN and the DMN. They further suggested that the latter could be used to develop a predictive model for insomnia progression (56). Similarly, Kim et al. (57) found that in PID patients, the FC strength between the VN and DMN was negatively correlated with sleep quality, and that VN activity changed following treatment. Specifically, their study revealed increased VN activity after taVNS treatment. Our results are consistent with these findings and further indicate that taVNS may exert its therapeutic effect on PID by modulating VN activity.

The hippocampus is a key brain region for regulating sleep, memory, and emotion. Dysfunction of the hippocampus often manifests in patients as anxiety, depression, and memory decline (58, 59). Stolicyn et al. (58) showed that PID patients exhibit abnormal function and volume in both the hippocampus and thalamus, suggesting that these alterations could serve as imaging markers for diagnosing insomnia. Zhang et al. (60) also found that insomnia can induce hippocampal inflammation and that inhibiting this inflammation may relieve insomnia symptoms. Given that taVNS can ameliorate hippocampal inflammation (59), our results suggest that taVNS may exert its therapeutic effect on PID by inhibiting inflammation and regulating hippocampal activity.

Regarding ANS activity, we found that the rMSSD, HF, and pNN50 in the treatment group were significantly higher during taVNS than in the control group during sham stimulation. These indicators reflect vagal tone, which is often characterized by low excitability coupled with sympathetic over-activity in PID patients (61). The observed increase in rMSSD and HF indicates that taVNS can enhance vagal activity. This finding aligns with previous research; for instance, Kang et al. (62) demonstrated that taVNS can modulate ANS activity to help treat related diseases, and Ma et al. (45) showed that vagus nerve stimulation improves sleep quality by modulating the ANS. Collectively, these results indicate that taVNS may exert therapeutic effects on PID by enhancing vagus nerve activity.

In addition, the efficacy of taVNS for PID varies considerably across individuals (18, 20, 63). Even for conditions approved by the U. S. Food and Drug Administration (FDA) for taVNS treatment, such as epilepsy and major depression, the response rate exhibits considerable fluctuation (21–23). For example, Roldán et al. (64) reported an effectiveness rate of 66% in patients with drug-resistant epilepsy treated with taVNS, while Rong et al. (65) observed that 54% of such patients experienced reduced seizure frequency and symptomatic relief after 24 weeks’ taVNS treatment. This variability is also evident with implanted vagus nerve stimulators. In a study that investigated implantable devices for drug-resistant epilepsy, Englot et al. (21) found that patients responded differently to identical stimulation parameters; those with greater HRV changes during VNS showed better efficacy than those with smaller alterations. Similarly, in neuroimaging research, Wu et al. (18) found that insomnia patients with differing responses to taVNS exhibited distinct brain activity patterns during stimulation.

These studies suggest that patients exhibit varying sensitivity to taVNS, which may influence its efficacy. Since fMRI and HRV measures can reflect an individual’s sensitivity and response to taVNS and show a correlation with clinical outcomes, we analyzed the associations among taVNS efficacy, HRV indicators, and pre-treatment mALFF values. This analysis aims to explore the underlying brain functional mechanisms of taVNS and to identify potential biomarkers for predicting treatment efficacy.

Our study found that the pre-treatment mALFF values of Cluster 4 (Precuneus_L, Cuneus_L, Superior occipital_L) and of the combined significant brain regions were correlated with taVNS efficacy. Additionally, the HF during taVNS treatment was correlated with the mALFF value of Cluster 4 (*p* < 0.015; Figures 8, 9).

The conchae cavity and the posterior wall of the external auditory canal constitute the afferent pathway of the vagus nerve (16, 66). The brain regions “Precuneus_L + Cuneus_L” belong to the DMN, while “Superior occipital_L + Cuneus_L” belong to the VN. Our results indicate that taVNS, by stimulating the transcutaneous auricular vagus nerve branches, transmits signals along the vagal pathway to modulate the activity of the DMN, VN, and SMN. This central modulation subsequently regulates ANS activity in PID patients, potentially outlining the mechanism through which taVNS treats PID. Furthermore, individual differences in the functional responsiveness of the DMN, VN, and SMN networks (particularly the DMN and VN) to ANS modulation during taVNS were correlated with variability in treatment efficacy. Additionally, baseline activity in the DMN and VN, along with HF parameters during stimulation, demonstrated potential as predictive biomarkers for taVNS efficacy.

### 4.1 Limitations

Our study has the following limitations: (1) We only conducted an randomized controlled trial study on the first 54 patients. In order to further demonstrate the efficacy of taVNS, we recruited another 46 patients underwent true taVNS treatment in the later stages, and the sample size of control group is insufficient. (2) Due to the relatively small sample size, we did not conduct a stratified study by age, as studies have shown that there may be differences in the state of the autonomic nervous system (ANS) among different age groups. (3) The treatment duration was short, at only 4 weeks; a comprehensive follow-up for understanding the mid- and long-term efficacy of taVNS was not performed.

Our study has the following limitations: (1) the initial phase was a randomized controlled trial involving only 54 patients. To further assess efficacy, we subsequently recruited an additional 46 patients who underwent true taVNS treatment; consequently, the sample size of the control group was insufficient; (2) owing to the relatively small sample size, we did not conduct an age-stratified analysis, even though studies suggest potential differences in autonomic nervous system (ANS) state across age groups; (3) the treatment duration was short (only four weeks), and no follow-up was conducted to evaluate the mid- or long-term efficacy of taVNS.

## Data Availability

The datasets generated and/or analyzed during the current study are not publicly available due to ethical restrictions and patient privacy concerns but are available from the corresponding author on reasonable request. The data contain sensitive personal health information, and public sharing would compromise participant confidentiality as guaranteed in our informed consent process and approved by the Ethics Committee of the Sichuan Academy of Medical Sciences, Sichuan Provincial Hospital. Requests to access the datasets should be directed to Xiao Wu, wuxiaozy@med.uestc.edu.cn.
